# p16 Mutation Spectrum in the Premalignant Condition Barrett's Esophagus

**DOI:** 10.1371/journal.pone.0003809

**Published:** 2008-11-27

**Authors:** Thomas G. Paulson, Patricia C. Galipeau, Lianjun Xu, Heather D. Kissel, Xiaohong Li, Patricia L. Blount, Carissa A. Sanchez, Robert D. Odze, Brian J. Reid

**Affiliations:** 1 Division of Human Biology, Fred Hutchinson Cancer Research Center, Seattle, Washington, United States of America; 2 Division of Public Health Sciences, Fred Hutchinson Cancer Research Center, Seattle, Washington, United States of America; 3 Department of General Surgery, Oregon Health & Sciences University, Portland, Oregon, United States of America; 4 Department of Medicine, University of Washington, Seattle, Washington, United States of America; 5 Department of Genome Sciences, University of Washington, Seattle, Washington, United States of America; 6 Department of Pathology, Brigham and Women's Hospital, Boston, Massachusetts, United States of America; Texas Tech University Health Sciences Center, United States of America

## Abstract

**Background:**

Mutation, promoter hypermethylation and loss of heterozygosity involving the tumor suppressor gene p16 (CDKN2a/INK4a) have been detected in a wide variety of human cancers, but much less is known concerning the frequency and spectrum of p16 mutations in premalignant conditions.

**Methods and Findings:**

We have determined the p16 mutation spectrum for a cohort of 304 patients with Barrett's esophagus, a premalignant condition that predisposes to the development of esophageal adenocarcinoma. Forty seven mutations were detected by sequencing of p16 exon 2 in 44 BE patients (14.5%) with a mutation spectrum consistent with that caused by oxidative damage and chronic inflammation. The percentage of patients with p16 mutations increased with increasing histologic grade. In addition, samples from 3 out of 19 patients (15.8%) who underwent esophagectomy were found to have mutations.

**Conclusions:**

The results of this study suggest the environment of the esophagus in BE patients can both generate and select for clones with p16 mutations.

## Introduction

p16 (CDKN2a/INK4a) (OMIM #600160) is a cyclin dependent kinase inhibitor that regulates cell cycle progression through the G1/S restriction point by binding to cyclin dependent kinases 4 and 6, preventing phosphorylation of the retinoblastoma protein [1], [2]. Germline mutations in p16 have been associated with familial melanoma syndromes [3], and somatic alterations in p16 have been detected in a wide variety of cancers [4], [5]. These alterations occur by multiple mechanisms, including mutation, loss of heterozygosity (LOH) and promoter hypermethylation. Given its role in modulating cell proliferation, it is not surprising that p16 alterations are one of the most common genetic/epigenetic alterations in cancer.

Barrett's esophagus (BE) is a premalignant condition in which the squamous epithelium that normally lines the esophagus is replaced with specialized intestinal epithelium as a result of chronic gastroesophageal reflux [6]. Patients with BE have a 30–40 fold increased risk of developing esophageal adenocarcinoma (EA), a cancer that has increased in incidence more than 600% over the past three decades (1972–2002) [7]–[9]. EA and esophageal squamous cell carcinoma (SCC) are the two major types of cancer that develop in the esophagus. In contrast to EA, SCC rates have declined in the US over the same time period [9]. Both types of esophageal cancer are thought to be promoted by environmental exposures (smoking and alcohol use for SCC, chronic gastroesophageal reflux for EA), but the molecular pathways involved with neoplastic progression in these two cancers are thought to be different [10]. A number of studies have examined the involvement of p16 mutations in esophageal cancer [4], [11]–[15], the majority of which have focused primarily upon SCC. Less is know concerning the frequency and spectrum of p16 mutations in EA, and very little about p16 mutations in BE. Here, we report p16 mutation detection and characterization in a prospective cohort study of 304 patients with BE and from 19 patients for which esophagectomy samples were available. We find p16 mutations can occur very early during neoplastic progression in BE, and the spectrum of mutation is consistent with that of oxidative damage that can be generated as a result of chronic reflux.

## Materials and Methods

### Patient characteristics and biopsy acquisition

Patients were enrolled in The Seattle Barrett's Esophagus Research Program, a dynamic cohort study that began in 1983. All participants in this study were recruited from this continuing program of cancer surveillance in which participants undergo periodic endoscopy with multiple biopsies following a standard protocol [16]. The study was approved by the Human Subjects Division of the University of Washington in 1983 and renewed annually thereafter with reciprocity from the Fred Hutchinson Cancer Research Center from 1993 to 2001. Since 2001, the study has been approved annually by the IRB of the FHCRC with reciprocity from the Human Subjects Division of the University of Washington. Flow-sorted samples from multiple biopsies of Barrett's epithelium were evaluated from 304 patients who at baseline endoscopy had BE without cancer. Of the 304 patients, 235 were male (77%) and 69 were female (23%). The mean age of the patients was 62.7 years (range 30.5 to 87.3) (Table 1). This study was conducted at a specialty research and referral center, and thus our cohort is considered a high-risk patient population. Biopsies were acquired at 2-cm intervals throughout the Barrett's segment using endoscopic mapping protocols described previously [16]–[19]. Normal gastric tissue served as a constitutive control for each patient. Patients were counseled regarding the risks and benefits of endoscopic surveillance and informed of potential alternative therapies, including surgery and endoscopic ablation, for high-grade dysplasia. Written informed consent for research use of biopsies and esophagectomy specimens was obtained from all patients in this study. Samples from 27 patients who had undergone esophagectomy were also examined for p16 mutation. Eight of these patients also had samples from a baseline endoscopy and were removed from analysis of frequency of mutation to prevent duplication of data.

**Table 1:** Cohort characteristics.

**Patients with endoscopic samples**	Male	235
	Female	69
	Average age	63 (range 30 to 87)
	Average # biopsies per patient	4.4
	**Diagnosis**	**Mutation frequency**
	High-grade dysplasia	12/61 (19.7%)
	Low-grade/Indefinite	27/153 (17.6%)
	Metaplasia	5/90 (5.5%)
	All	44/304 (14.5%)
**Patients with esophagectomy samples**	Male	18
	Female	1
	Average age	67 (range 43 to 93)
	Average # biopsies per patient	5.4
	**Diagnosis**	**Mutation frequency**
	EA	1/13 (6.25%)
	High-grade dysplasia	1/3 (33.3%)
	Low-grade/Indefinite	1/1 (100.0%)
	Metaplasia	0/0 (NA)
	Unknown	0/2 (0.0%)
	All	3/19 (15.8%)

### Histology

Endoscopic biopsies were processed and interpreted for grade of dysplasia. Mutation at p16 was assessed in biopsies taken at levels adjacent to those used for histology and patients were classified according to the maximum histologic grade of dysplasia in any biopsy, as described previously [16], [17], [19]. Of the 304 patients, 90 were negative for dysplasia, 95 were indefinite for dysplasia, 58 had low-grade dysplasia range (LGD), and 61 had high-grade dysplasia (HGD) (Table 1). The maximum histologic grade of the samples from the esophagectomies ranged from unknown (n = 2) to low-grade/indefinite for dysplasia (1), high-grade dysplasia (8), up through EA (16). Histologic or p16 mutation data from previous endoscopies from many of these patients were not available; because of this, data from the esophagectomy specimens was analyzed separately from that obtained from endoscopic biopsies.

### Flow Cytometric Sorting and DNA Extraction

Barrett's epithelial cell populations were purified from endoscopic biopsies and esophagectomy samples by means of Ki67/DNA content flow sorting of diploid G1, 4N, and aneuploid cell populations on a Coulter Elite ESP cell sorter, as described previously [20]–[22]. This process enriches for epithelial cell (BE) populations and removes underlying genotypically normal stromal cells, allowing less ambiguous detection and characterization of mutations.

### DNA Sequencing

Evaluation of mutations in exon 2 of the p16 gene was performed on an aliquot of genomic DNA that had undergone whole genome amplification (PEP) [23] as previously described [24]. We sequenced exon 2 of p16 since the vast majority of p16 mutations reported in the literature lie within this exon. Sequencing was performed using BigDye Terminator cycle sequencing (Applied Biosystems, Foster City, CA) on an ABI 377 DNA sequencer. Wild type sequences for each patient were confirmed using constitutive (gastric) samples. All mutations were confirmed by at least two independent PCR and sequencing reactions, and in cases of ambiguity, by direct sequencing of genomic DNA. The mutation sequence for 2 patients have been previously reported [24], [25] and the frequency of p16 mutations, but not the sequences, for a subset of the patients in this study (N = 107) was reported in a previous publication [24].

### LOH status and deletion status

LOH status was determined as described previously [21], [26]. The LOH status of the patients in this study has been presented previously [24], [25], [27], [28], but the associations between LOH and p16 mutation have not been presented previously. Deletions at the p16 locus were determined using array CGH of BAC clones in and around the p16 locus, which are described in detail elsewhere (Paulson, et al, submitted). LOH was further defined as copy neutral (loss of one allele, but no change in copy number) or copy loss LOH (loss of one allele due to deletion of p16 sequence). Deletion data were available for 105 samples from 65 patients for analysis of copy neutral and copy loss LOH.

### Clonal ordering

The order of genetic events can be determined by comparing which events are present in biopsies from patients in which both events are observed [29]. For events A and B, there are 4 possible relationships: A precedes B, B precedes A, A and B occur together, or A and B are independent of each other.

### Statistical analyses

Assessment of a trend for more p16 mutations in samples from patients with more advanced histologic diagnoses was performed using the Cochran-Armitage trend test. Frequencies of p16 mutations in different subgroups were compared using Fischer's exact test.

## Results

**Figure 1:**
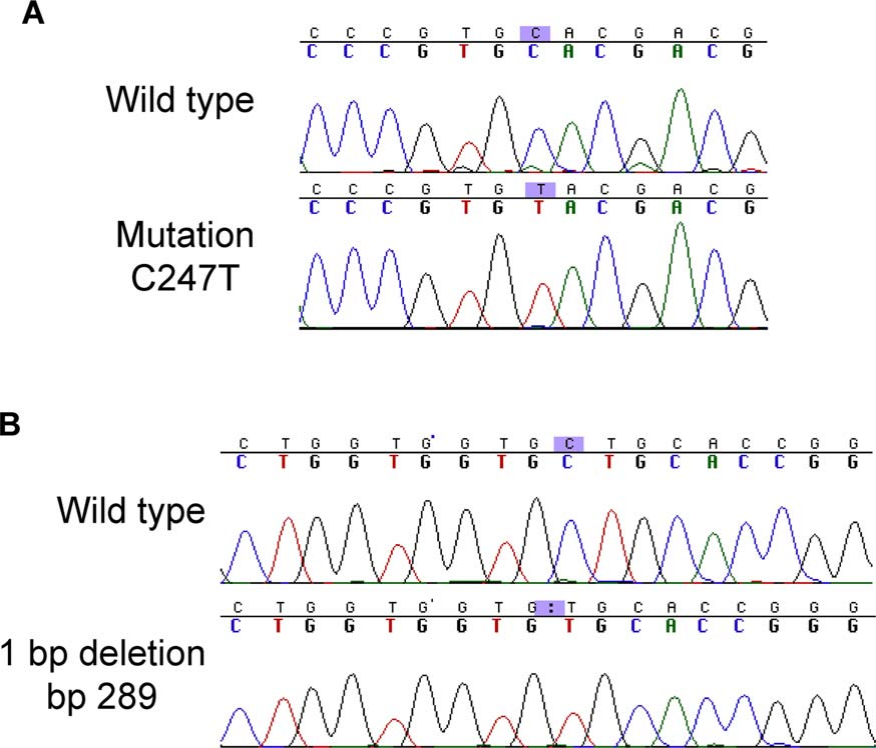
Examples of p16 mutations in Barrett's esophagus. A) C to T transition at basepair 247. B) 1 basepair deletion at nucleotide 289.

**Figure 2:**
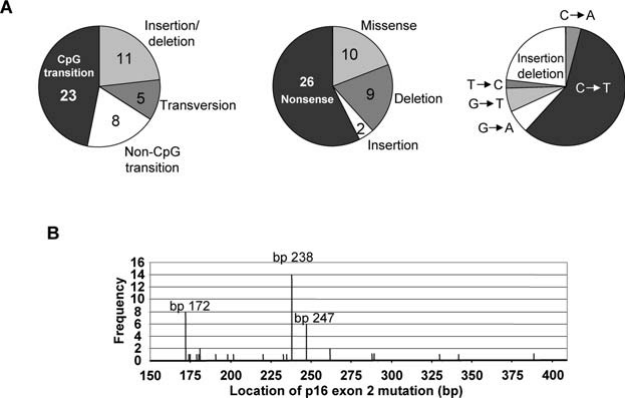
Spectrum of p16 mutations in patients with Barrett's esophagus. A. Types of mutations observed in BE patients. Number of events are indicated. B. Location, by base pair and frequency (number of events), of p16 mutations in BE patients.

We evaluated 1346 biopsies from 304 patients with BE (average of 4 per patient, range 1 to 20) having diagnoses ranging from metaplasia negative for dysplasia to HGD (Table 1). Forty-four of the 304 patients (14.5%) had a total of 47 mutations in endoscopic biopsies from the Barrett's segment (Figure 1). Three patients had two different p16 mutations that were located in spatially distinct regions of the Barrett's segment. No germline mutations were detected in the constitutive samples. Fewer alterations were found in patients with metaplasia alone and there was a significant trend towards a higher frequency of alterations with increasing histologic grade (p = 0.009). The spectrum of these mutations is indicated in Figure 2 and Table 2. Sixty percent of the mutations occurred at three sites–bp 172 (R58X), bp 238 (R80X) and bp 247 (H83Y)–and were all C→T transitions. Transitions at CpG sites (49%) and insertions/deletion (23%) made up the majority of the observed mutations. Of the 36 point mutations, 88% (32/36) resulted in either a conservative amino acid change or no change in the coding sequence of p14 ARF. Seven patients of the 27 having samples from esophagectomy specimens had mutations in p16 (Table 3). Some patients (n = 8) had samples available both from baseline endoscopy as well as from an esophagectomy: of these 8 patients, all those that had a p16 mutation present in the esophagectomy specimen (n = 4) had the same mutation(s) present at baseline endoscopy. Samples from 3 of the remaining 19 esophagectomy samples (15.8%) contained p16 mutations.

**Table 2:** Mutations in p16/CDKN2a detected in patients with Barrett's esophagus.

Alteration*	Diagnosis	p14ARF status	Percent of BE segment
172C>T^d^	HGD	Pro to Leu	29
172C>T	Metaplasia	Pro to Leu	50
172C>T	HGD	Pro to Leu	43
172C>T	IND/LGD	Pro to Leu	67
172C>T	HGD	Pro to Leu	33
172C>T	HGD	Pro to Leu	100
172C>T^a^^,^ d	HGD	Pro to Leu	No data
172C>T^b^	HGD	Pro to Leu	33
174_175del2	IND/LGD	Frameshift	100
175_221del47	IND/LGD	Frameshift	25
179C>A	IND/LGD	No alteration	100
179_183dup5	IND/LGD	Frameshift	100
182_283del102	Metaplasia	deletion	100
181G>T	Metaplasia	Gly to Val	67
191_205del15	HGD	deletion	100
198_343del146	IND/LGD	deletion/frameshift	100
202G>A	IND/LGD	Arg to His	50
220G>A	IND/LGD	Arg to Gln	75
233_234del2^c^	IND/LGD	Frameshift	43
235_245del11	IND/LGD	Frameshift	100
238C>T	Metaplasia	Pro to Leu	100
238C>T^d^	HGD	Pro to Leu	50
238C>T	IND/LGD	Pro to Leu	40
238C>T	Metaplasia	Pro to Leu	100
238C>T	IND/LGD	Pro to Leu	100
238C>T	IND/LGD	Pro to Leu	33
238C>T	IND/LGD	Pro to Leu	17
238C>T	IND/LGD	Pro to Leu	17
238C>T	IND/LGD	Pro to Leu	100
238C>T	IND/LGD	Pro to Leu	100
238C>T	IND/LGD	Pro to Leu	50
238_254del17	IND/LGD	Frameshift	100
238C>T	IND/LGD	Pro to Leu	100
238C>T	HGD	Pro to Leu	25
247C>T^c^	IND/LGD	Ala to Val	14
247C>T	IND/LGD	Ala to Val	50
247C>T	HGD	Ala to Val	100
247C>T	IND/LGD	Ala to Val	80
247C>T	IND/LGD	Ala to Val	25
247C>T	HGD	Ala to Val	100
262G>T	IND/LGD	Gly to Val	100
262G>T	IND/LGD	Gly to Val	25
289delC^d^	HGD	Frameshift	33
290 T>C^b^	HGD	No alteration	33
330 G>A	IND/LGD	Gly to Arg	100
323_341dup19	IND/LGD	Frameshift	50
387C>A^a^^,^ d	HGD	Pro to Thr	100

a,b,c indicates three patients each having two distinct alterations found at different levels in the esophagus.

d indicates mutation that was also found in esophagectomy specimen from the same patient.

* Reference sequence used was NM_000077; nucleotide numbering is as found in The CDKN2A Database[4]. Diagnosis is at baseline endoscopy.

** Indicates the percentage of the esophagus having Barrett's epithelium that contained the mutation.

**Table 3:** Mutations in p16/CDKN2a detected in esophagectomy patients.

Alteration	Diagnosis	p14ARF status	Percent of BE segment
173dupC	EA	Frameshift	NA
197A>C	IND/LGD	No alteration	NA
329G>A	HGD	No alteration	NA

Headings are same as in Table 2. Diagnosis is at the time of the esophagectomy.

Consistent with earlier studies [24], [25], clonal cell populations with p16 mutations were found to have expanded in the Barrett's segment. Among patients whose Barrett's segment length was ≥2 cm, p16 mutations extended over an average of 66.4% of the Barrett's epithelium, including cases in which the same mutation was detected in 6 biopsies across 12 cm of esophageal mucosa. These results are similar to those obtained when using 9p21 LOH as a measure of clonality, in which clones with the same LOH patterns were found over an average of 55% of the esophagus, to a maximum of 100% of a 17 cm segment.

p16 mutation represents one of the two hits required to inactivate the gene, along with copy neutral LOH, deletion/copy loss LOH, or promoter hypermethylation; therefore, we examined the associations between p16 mutation, LOH and deletion in patients with BE. We found no statistical difference between the frequency of copy neutral and copy loss LOH (51% vs. 49%, respectively). However, we found copy neutral LOH was much more common in samples with a p16 mutation (7/8, 87.5%) than in samples without a p16 mutation (20/45, 44.4%) (p = 0.05), and that p16 mutation was found in samples with copy neutral LOH significantly more often than with copy loss LOH (7/27 vs 1/26, p = 0.05). Finally, we examined the order in which p16 mutation and LOH (either type) occurred in this BE cohort. In 18 evaluable patients in which both LOH and mutation were found, we found independent LOH and mutation events in 6 patients, LOH occurred before mutation in 8 patients and mutation before LOH in 4 patients, suggesting that when mutation and LOH both happen, there is no preferential order.

## Discussion

Previous studies examining the frequency of p16 mutation in esophageal cancer have focused primarily upon SCC in surgical resections, with reported frequencies of mutation ranging from 0% to 52%, with most studies reporting 15–20% [4], [14], [30]. We found 15.8% of esophagectomy samples to have p16 mutations, similar to the frequency in BE patients (14.5%) . Our study provides the most extensive examination to date of p16 mutations in a premalignant tissue (BE). p16 mutations were detected at all histologic grades, indicating they can develop as very early events in neoplastic progression of BE. Since p16 can be inactivated by multiple mechanisms, including methylation and LOH, analysis of cancer risk associated with p16 mutation alone was not possible in this study. However, we have evaluated a cohort of 138 patients for whom mutation, LOH and methylation were evaluated and found no difference in cancer risk between patients with p16 mutation alone vs. methylation or LOH alone (TG Paulson, unpublished observations). As well, in a prospective study of multiple variables influencing risk of progression to EA, p16 mutation alone was not found to have significant predictive value [27]. These findings support the idea that loss of p16 function (and possibly other genes in the 9p21 chromosomal region), rather than the mechanism of its inactivation, is the important step in the development of EA.

Mutations at the p16 codons altered in BE have been observed in a variety of cancers, including melanoma, bladder, oral SCC and NSCLC [31]. Two of the three most common mutations observed in BE, at amino acids 58 and 80 (bp 172 and 238, respectively), result in a truncated protein, and the third most common BE alteration, H83Y (bp 247), has been shown to be defective in biochemical analyses [32], [33]. All of the non-insertion-deletion mutations conserved the open reading frame for ARF, suggesting specific targeting of the p16 locus for inactivation, a phenomenon reported in other tumor types as well [34].

The p16 mutation spectrum we observed is consistent with that mediated by reactive oxygen and nitric oxide species generated in response to inflammation and tissue damage [35]. Direct exposure to acid and bile reflux and the subsequent chronic inflammation are two potential sources of oxidative damage characteristic of patients with BE [37]–[39].The higher frequency of p16 mutation at later stages of BE and the fact that genetic alterations that have been shown to occur later during neoplastic progression in BE, such as p53 mutations, also display a similar mutation spectrum [36], suggest that the ROS generated by tissue damage and inflammation continue to act through neoplastic progression.

Oxidative damage of DNA, proteins and lipids is hypothesized to play an etiologic role in many types of cancers, particularly those characterized by chronic inflammation. In the esophagus, both *in vitro* [40] and *in vivo* [41], [42] studies indicate exposure to gastroduodenal reflux results in measurable oxidative damage to DNA. As well, p16 alterations have been observed in animal models in response to oxidative stress [43], [44], and it has been proposed that oxidative damage may be responsible for the loss of heterozygosity frequently observed at multiple chromosomal loci in BE [45], [46]. Oxidative stress can also induce p16 mediated senescence (reviewed in [34]); thus, the oxidative damage induced by gastroduodenal reflux provides both a mechanism for generating genetic alterations as well as a selective pressure for loss of wt p16 function.

To our knowledge, this is the largest study of p16 mutation spectrum yet reported in a premalignant condition. The study was based on a cohort design and all biopsies were obtained prospectively using standardized protocols. The characteristics of our cohort, including age, gender, and Barrett's segment length are comparable to studies from specialty centers [47], [48]. Our study was performed in a tertiary referral center and our research cohort therefore has a higher percentage of patients with a diagnosis of dysplasia than the general BE population; however, this is unlikely to have affected the p16 mutation spectrum reported here because we also detected p16 mutations in patients without high-grade dysplasia, indicating p16 mutations can occur very early during neoplastic progression. The observation that frequency of mutation increased with histologic grade suggests these patients may have experienced a longer or more intense exposure to oxidative damage induced by reflux.

All of the data obtained in this study are consistent with Knudsen's two-hit model for inactivating tumor suppressor genes [49]. LOH is the predominant mechanism of inactivation for p16, occurring in almost 60% of patients with BE, compared to 14.5% with mutation. This may represent the fact that the genetic mechanisms that result in LOH (mitotic recombination, non-disjunction and reduplication, and/or deletion) are more common than the development of point mutations. Alternatively, LOH events may involve additional genes (e.g., p14ARF and p15), leading to a greater selective advantage over clones containing mutations.

We examined only a single biopsy every 2 cm from each patient's Barrett's segment, making it possible that the frequencies of mutation we observe are underestimates of the true value. However, since clones with p16 mutations were observed to have covered an average of 66% of the Barrett's segment, it is unlikely that many alterations were missed. However, it is possible we missed rare mutations that occurred in exon 1 of p16. The frequency of p16 mutation we detected is higher than previously reported in EA or in BE found in surgical resections [11], [15], [30], [50]–[53]. Our use of a flow cytometric purification of proliferating epithelial cells may explain this higher frequency since it eliminates possible masking of a mutation signal by the presence of genotypically normal stromal cells. The results from this study provide strong evidence that alterations in p16 occur early during neoplastic progression in patients with BE and that the physiological consequences of chronic gastroduodenal reflux are the likely causes of these alterations.

## References

[1] Sherr CJ, McCormick F (2002). The RB and p53 pathways in cancer.. Cancer Cell.

[2] Ruas M, Peters G (1998). The p16INK4a/CDKN2A tumor suppressor and its relatives.. Biochim Biophys Acta.

[3] Pho L, Grossman D, Leachman SA (2006). Melanoma genetics: a review of genetic factors and clinical phenotypes in familial melanoma.. Curr Opin Oncol.

[4] Murphy JA, Barrantes-Reynolds R, Kocherlakota R, Bond JP, Greenblatt MS (2004). The CDKN2A database: Integrating allelic variants with evolution, structure, function, and disease association.. Hum Mutat.

[5] Rocco JW, Sidransky D (2001). p16(MTS-1/CDKN2/INK4a) in cancer progression.. Exp Cell Res.

[6] Phillips RW, Wong RK (1991). Barrett's esophagus. Natural history, incidence, etiology, and complications.. Gastroenterol Clin North Am.

[7] Pohl H, Welch HG (2005). The role of overdiagnosis and reclassification in the marked increase of esophageal adenocarcinoma incidence.. J Natl Cancer Inst.

[8] Holmes RS, Vaughan TL (2007). Epidemiology and pathogenesis of esophageal cancer.. Semin Radiat Oncol.

[9] Brown LM, Devesa SS (2002). Epidemiologic trends in esophageal and gastric cancer in the United States.. Surg Oncol Clin N Am.

[10] Lin J, Beerm DG (2004). Molecular biology of upper gastrointestinal malignancies.. Semin Oncol.

[11] Esteve A, Martel-Planche G, Sylla BS, Hollstein M, Hainaut P (1996). Low frequency of p16/CDKN2 gene mutations in esophageal carcinomas.. Int J Cancer.

[12] Giroux MA, Audrezet MP, Metges JP, Lozac'h P, Volant A (2002). Infrequent p16/CDKN2 alterations in squamous cell carcinoma of the oesophagus.. Eur J Gastroenterol Hepatol.

[13] Gamieldien W, Victor TC, Mugwanya D, Stepien A, Gelderblom WC (1998). p53 and p16/CDKN2 gene mutations in esophageal tumors from a high-incidence area in South Africa.. Int J Cancer.

[14] Smeds J, Berggren P, Ma X, Xu Z, Hemminki K (2002). Genetic status of cell cycle regulators in squamous cell carcinoma of the oesophagus: the CDKN2A (p16(INK4a) and p14(ARF) ) and p53 genes are major targets for inactivation.. Carcinogenesis.

[15] Zhou X, Tarmin L, Yin J, Jiang HY, Suzuki H (1994). The MTS1 gene is frequently mutated in primary human esophageal tumors.. Oncogene.

[16] Reid B, Blount P, Rabinovitch P (2003). Biomarkers in Barrett's Esophagus: A Guideline for Clinicians.. Gastrointest Endosc Clin N Am.

[17] Reid BJ, Weinstein WM, Lewin KJ, Haggitt RC, VanDeventer G (1988). Endoscopic biopsy can detect high-grade dysplasia or early adenocarcinoma in Barrett's esophagus without grossly recognizable neoplastic lesions.. Gastroenterology.

[18] Levine DS, Haggitt RC, Blount PL, Rabinovitch PS, Rusch VW (1993). An endoscopic biopsy protocol can differentiate high-grade dysplasia from early adenocarcinoma in Barrett's esophagus [see comments].. Gastroenterology.

[19] Reid BJ, Blount PL, Feng Z, Levine DS (2000). Optimizing endoscopic biopsy detection of early cancers in Barrett's high-grade dysplasia.. Am J Gastroenterol.

[20] Blount PL, Galipeau PC, Sanchez CA, Neshat K, Levine DS (1994). 17p allelic losses in diploid cells of patients with Barrett's esophagus who develop aneuploidy.. Cancer Res.

[21] Paulson TG, Galipeau PC, Reid BJ (1999). Loss of heterozygosity analysis using whole genome amplification, cell sorting, and fluorescence-based PCR.. Genome Res.

[22] Reid BJ, Sanchez CA, Blount PL, Levine DS (1993). Barrett's esophagus: cell cycle abnormalities in advancing stages of neoplastic progression [see comments].. Gastroenterology.

[23] Zhang L, Cui X, Schmitt K, Hubert R, Navidi W (1992). Whole genome amplification from a single cell: implications for genetic analysis.. Proc Natl Acad Sci U S A.

[24] Wong DJ, Paulson TG, Prevo LJ, Galipeau PC, Longton G (2001). p16(INK4a) lesions are common, early abnormalities that undergo clonal expansion in Barrett's metaplastic epithelium.. Cancer Res.

[25] Barrett MT, Sanchez CA, Galipeau PC, Neshat K, Emond M (1996). Allelic loss of 9p21 and mutation of the CDKN2/p16 gene develop as early lesions during neoplastic progression in Barrett's esophagus.. Oncogene.

[26] Galipeau PC, Cowan DS, Sanchez CA, Barrett MT, Emond MJ (1996). 17p (p53) allelic losses, 4N (G2/tetraploid) populations, and progression to aneuploidy in Barrett's esophagus.. Proc Natl Acad Sci U S A.

[27] Galipeau PC, Li X, Blount PL, Maley CC, Sanchez CA (2007). NSAIDs modulate CDKN2A, TP53, and DNA content risk for progression to esophageal adenocarcinoma.. PLoS Med.

[28] Galipeau PC, Prevo LJ, Sanchez CA, Longton GM, Reid BJ (1999). Clonal expansion and loss of heterozygosity at chromosomes 9p and 17p in premalignant esophageal (Barrett's) tissue.. J Natl Cancer Inst.

[29] Blount PL, Meltzer SJ, Yin J, Huang Y, Krasna MJ (1993). Clonal ordering of 17p and 5q allelic losses in Barrett dysplasia and adenocarcinoma.. Proc Natl Acad Sci U S A.

[30] Muzeau F, Flejou JF, Belghiti J, Thomas G, Hamelin R (1997). Infrequent microsatellite instability in oesophageal cancers.. Br J Cancer.

[31] Foulkes WD, Flanders TY, Pollock PM, Hayward NK (1997). The CDKN2A (p16) gene and human cancer.. Mol Med.

[32] Yarbrough WG, Buckmire RA, Bessho M, Liu ET (1999). Biologic and biochemical analyses of p16(INK4a) mutations from primary tumors.. J Natl Cancer Inst.

[33] Greenblatt MS, Beaudet JG, Gump JR, Godin KS, Trombley L (2003). Detailed computational study of p53 and p16: using evolutionary sequence analysis and disease-associated mutations to predict the functional consequences of allelic variants.. Oncogene.

[34] Gil J, Peters G (2006). Regulation of the INK4b-ARF-INK4a tumour suppressor locus: all for one or one for all.. Nat Rev Mol Cell Biol.

[35] Evans MD, Dizdaroglu M, Cooke MS (2004). Oxidative DNA damage and disease: induction, repair and significance.. Mutat Res.

[36] Prevo LJ, Sanchez CA, Galipeau PC, Reid BJ (1999). p53-mutant clones and field effects in Barrett's esophagus.. Cancer Res.

[37] Bernstein H, Bernstein C, Payne CM, Dvorakova K, Garewal H (2005). Bile acids as carcinogens in human gastrointestinal cancers.. Mutat Res.

[38] Hussain SP, Hofseth LJ, Harris CC (2003). Radical causes of cancer.. Nat Rev Cancer.

[39] Wiseman H, Halliwell B (1996). Damage to DNA by reactive oxygen and nitrogen species: role in inflammatory disease and progression to cancer.. Biochem J.

[40] Dvorak K, Fass R, Dekel R, Payne CM, Chavarria M (2006). Esophageal acid exposure at pH</ = 2 is more common in Barrett's esophagus patients and is associated with oxidative stress.. Dis Esophagus.

[41] Wetscher GJ, Hinder RA, Klingler P, Gadenstatter M, Perdikis G (1997). Reflux esophagitis in humans is a free radical event.. Dis Esophagus.

[42] Liu L, Ergun G, Ertan A, Woods K, Sachs I (2003). Detection of oxidative DNA damage in oesophageal biopsies of patients with reflux symptoms and normal pH monitoring.. Aliment Pharmacol Ther.

[43] Hiroyasu M, Ozeki M, Kohda H, Echizenya M, Tanaka T (2002). Specific allelic loss of p16 (INK4A) tumor suppressor gene after weeks of iron-mediated oxidative damage during rat renal carcinogenesis.. Am J Pathol.

[44] Tanaka T, Iwasa Y, Kondo S, Hiai H, Toyokuni S (1999). High incidence of allelic loss on chromosome 5 and inactivation of p15INK4B and p16INK4A tumor suppressor genes in oxystress-induced renal cell carcinoma of rats.. Oncogene.

[45] Turker MS, Gage BM, Rose JA, Elroy D, Ponomareva ON (1999). A novel signature mutation for oxidative damage resembles a mutational pattern found commonly in human cancers.. Cancer Res.

[46] Turner DR, Dreimanis M, Holt D, Firgaira FA, Morley AA (2003). Mitotic recombination is an important mutational event following oxidative damage.. Mutat Res.

[47] Cameron AJ, Lomboy CT (1992). Barrett's esophagus: age, prevalence, and extent of columnar epithelium.. Gastroenterology.

[48] O'Connor JB, Falk GW, Richter JE (1999). The incidence of adenocarcinoma and dysplasia in Barrett's esophagus: report on the Cleveland Clinic Barrett's Esophagus Registry.. Am J Gastroenterol.

[49] Knudson AG (1971). Mutation and cancer: statistical study of retinoblastoma.. Proc Natl Acad Sci U S A.

[50] Roncalli M, Bosari S, Marchetti A, Buttitta F, Bossi P (1998). Cell cycle-related gene abnormalities and product expression in esophageal carcinoma.. Lab Invest.

[51] Hardie LJ, Darnton SJ, Wallis YL, Chauhan A, Hainaut P (2005). p16 expression in Barrett's esophagus and esophageal adenocarcinoma: association with genetic and epigenetic alterations.. Cancer Lett.

[52] Vieth M, Schneider-Stock R, Rohrich K, May A, Ell C (2004). INK4a-ARF alterations in Barrett's epithelium, intraepithelial neoplasia and Barrett's adenocarcinoma.. Virchows Arch.

[53] Gonzalez MV, Artimez ML, Rodrigo L, Lopez-Larrea C, Menendez MJ (1997). Mutation analysis of the p53, APC, and p16 genes in the Barrett's oesophagus, dysplasia, and adenocarcinoma.. J Clin Pathol.

